# Long-acting HIV pre-exposure prophylaxis integrated with community-based sexual and reproductive health services in South Africa (LAPIS): study protocol for a hybrid (1a) cluster randomised controlled phase 3B trial of effectiveness and implementation

**DOI:** 10.1186/s12889-025-24889-1

**Published:** 2026-01-20

**Authors:** Jacob Busang, Thembelihle Zuma, Natsayi Chimbindi, Nqobile Ngoma, Carina Herbst, Nonhlanhla Okesola, Jaco Dreyer, Theresa Smit, Kristien Bird, Lucky Mtolo, Ngundu Behuhuma, Limakatso Lebina, Cheryl Hendrickson, Jacqui Miot, Willem Hanekom, Kobus Herbst, Janet Seeley, Andrew Copas, Kathy Baisley, Maryam Shahmanesh, LAPIS Study Team, Manono Luthuli, Siphesihle Hlongwane, Sinakekelwe Nkwanyana, Phindile Khanyile, Dumisile Mthethwa, Lindiwe Sibiya, Minenhle Dlamini, Cyril Thwala, Samkelisiwe Ngubane, Priscilla Danisa, Sithembile Msane, Mthobisi Zikhali, Eva Ssozi, Siyabonga Dubazana, Ashley Jalazi, Zama Nkalane

**Affiliations:** 1 Africa Health Research Institute, Mtubatuba, KwaZulu-Natal South Africa; 2 University College London, Institute for Global Health, 3rd Floor Mortimer Market Centre, Capper Street, London, WC1E 6JP UK; 3 University of KwaZulu-Natal, Durban, South Africa; 4 School of Medicine, Faculty of Health Sciences, University of Pretoria, Pretoria, South Africa; 5 University of the Witwatersrand, Johannesburg, South Africa; 6 Health Economics and Epidemiology Research Office, Wits Health Consortium, Johannesburg, South Africa; 7 Department of Medical Microbiology, Academic Medical Center, University of Amsterdam, Amsterdam, Netherlands; 8 DSI-SAMRC South African Population Research Infrastructure Network, South African Medical Research Council, Durban, South Africa; 9 London School of Hygiene & Tropical Medicine, London, UK

**Keywords:** Long-acting PrEP, Injectable cabotegravir, Dapivirine vaginal ring, Adolescents and young adults, Peer support, Community-based sexual and reproductive health services, Pragmatic trial

## Abstract

**Background:**

Barriers and challenges associated with daily oral HIV pre-exposure prophylaxis (PrEP) contribute to poor uptake, low retention, and adherence rates among youth. Offering a choice of PrEP modalities integrated with peer support and delivered through community-based sexual and reproductive health (SRH) services will overcome these challenges. We describe the design of a trial to evaluate this approach at a population level.

**Methods:**

We are conducting a type 1a hybrid effectiveness, phase 3B, cluster randomised controlled trial (LAPIS) to evaluate the effectiveness and implementation of offering PrEP modality choices through community-based SRH services amongst youth aged 15–30 years living in rural Kwa-Zulu Natal, South Africa. LAPIS is nested within *Thetha nami ngithethe nawe* (Let’s Talk), an ongoing stepped-wedge trial with two periods investigating the effectiveness, implementation, and cost effectiveness of peer-led social mobilisation into decentralised integrated HIV and SRH services on the population prevalence of sexually transmissible HIV amongst youth. In the second period of *Thetha nami*, 40 trial clusters were randomised 1:1 to receive either a choice of PrEP modalities (oral PrEP, long-acting PrEP, i.e., two-monthly injectable cabotegravir (CAB LA) or dapivirine vaginal ring and HIV post-exposure prophylaxis [PEP] packs) or enhanced standard of care (ESoC) with oral PrEP only. All trial clusters are supported by peer navigators offering peer support and visited monthly by a mobile nurse-led clinic offering adolescent and youth-friendly HIV and SRH services. There are two primary outcomes: (1) effective uptake of PrEP or PEP, and (2) retention on PrEP, defined as attending at least one follow-up appointment after PrEP/PEP initiation, which are based on clinic data. Implementation outcomes are assessed using a mixed-methods and process evaluation following the RE-AIM (Reach, Effectiveness, Adoption, Implementation, and Maintenance) framework.

**Discussion:**

LAPIS is a pragmatic trial to evaluate the addition of long-acting PrEP modalities to daily oral PrEP within community-based SRH services. By offering PrEP choices, LAPIS adopts a person-centred approach to improve adherence and retention among youth, including hidden key populations. Findings will provide insights into the real-world implementation of CAB-LA.

**Trial registration:**

ClinicalTrials.gov Identifier—NCT06250504. Registered: 01 February 2024.

**Supplementary Information:**

The online version contains supplementary material available at 10.1186/s12889-025-24889-1.

## Introduction

South Africa continues to experience high HIV incidence, with approximately 160,000 new infections in 2022, and young people aged 15–24 years accounting for 35% of these new infections [1]. This high HIV incidence occurs in the context of the largest antiretroviral therapy (ART) program in the world, offering effective biomedical interventions, such as freely accessible HIV testing, universal test and treat (UTT) programs, and HIV pre-exposure prophylaxis (PrEP). Despite a decline in HIV prevalence among youth in South Africa, HIV incidence remains high among adolescent girls and young women (AGYW), particularly amongst young women aged 20–24 in rural KwaZulu-Natal (KZN) [2]. The overall decline is not fast enough to meet the UNAIDS 2030 targets which entails that 95% of people living with HIV know their HIV status, 95% of people who know their status are receiving HIV treatment, and 95% of people on treatment are virally suppressed.

Recent advances in biomedical HIV prevention tools have significantly enhanced HIV prevention efforts. These tools include HIV point-of-care tests (POCT) and self-tests, the use of daily oral PrEP and long-acting injectable PrEP with cabotegravir (CAB-LA), twice-yearly lenacapavir, and ART for HIV treatment, which eliminates onward transmission of the virus [3–5]. However, the impact of biomedical prevention such as PrEP depends on its uptake and consistent use by those at greatest risk. This has proven challenging, particularly for AGYW, who often have inaccurate risk perceptions [6] and face perceived and actual barriers in accessing PrEP through primary health care (PHC) clinics [7]. Retention on oral PrEP also remains a significant issue. In a previous study we found that whilst the majority (> 90%) of those offered PrEP initiate oral PrEP, less than 20% remain on PrEP for at least six months after initiation [8]. Among those who continue, 70% are young men at risk of HIV. One-third of users discontinue PrEP because they no longer perceive themselves at risk, while others discontinue due to the challenges of daily tablet intake, stigma, and misconceptions about PrEP in the community, as well as the association of oral PrEP and rattling pill bottles with HIV antiretrovirals. These barriers among young people have also been reported elsewhere [9–11].

Individual randomised trials among at-risk cisgender men who have sex with men (MSM) and at-risk transgender women who have sex with men (HPTN 083) [12], as well as cisgender women (HPTN 084) [13] showed that CAB-LA was safe and more effective than oral PrEP, with the efficacy driven almost entirely by adherence. Repeated surveys and qualitative studies in our setting show a preference for long acting and injectable PrEP among AYA (*unpublished data*). Therefore, we propose adding the choice of long-acting injectable PrEP to the community-based integrated HIV and sexual and reproductive health (SRH) services.

A body of evidence supports the effectiveness of community-based HIV care in increasing demand for SRH services and thus reducing sexually transmitted HIV [14–16]. Integrating community-based approaches with broader psychosocial care enables social networks and norms that promote HIV care, thereby contributing to sustainable development goals [17, 18]. Building on this evidence, we have established *Thetha nami ngithethe nawe* (Let’s Talk in isiZulu), a large implementation trial of community-based integrated SRH services with oral PrEP [19]. *Thetha nami ngithethe nawe (Thetha nami* for short*)* is a stepped-wedge cluster randomised controlled trial (SW-cRCT) consisting of peer support for PrEP demand creation and adolescent youth-friendly integrated HIV and SRH mobile services in rural South Africa, in an area with a high burden of HIV. This trial is targeting 15–30-year-old AYA living in uMkhanyakude district, KwaZulu-Natal, and aims to investigate the effectiveness, implementation, and cost-effectiveness of peer-led social mobilisation into decentralised, integrated HIV SRH services. The trial commenced enrolment in June 2022.

We are using the *Theta nami* platform to evaluate the addition of choice of long-acting HIV PrEP integrated with community-based SRH services (LAPIS). We hypothesise that offering a choice of long-acting PrEP, such as the two-monthly injectable cabotegravir, in addition to the community-based oral PrEP and SRH will overcome the adherence, retention, and disclosure challenges of oral PrEP and lead to a population-level effect on sexually transmissible HIV.

## Objectives

### Main trial objective

The overarching goal of the LAPIS trial is to evaluate the effectiveness of real-world implementation of offering a choice of oral PrEP, long-acting PrEP with injectable cabotegravir, dapivirine vaginal ring (DapiRing) or post-exposure prophylaxis-in-pocket (PEP-in-pocket) within community-based integrated HIV and SRH services on PrEP uptake and retention, and population prevalence of sexually transmissible HIV amongst adolescents and young adults in rural KZN, South Africa.

The research objectives are:

1. To measure the effectiveness of offering a choice of oral or long-acting injectable cabotegravir (CAB-LA) for PrEP and the availability of DapiRing and PEP-in-pocket on increasing effective uptake, retention and adherence to PrEP, and reducing sexually transmissible HIV and HIV incidence in young people aged 15–30 in rural South Africa.
2. To understand real-world implementation:
  2.1. To explore the acceptability, preference, and reach of CAB-LA from the perspective of young people aged 15–30 and their communities in rural South Africa.
  2.2. To understand the feasibility, affordability, and scalability of delivering CAB-LA through community-based PrEP with SRH.
  2.3. To identify implementation challenges and practical solutions for CAB-LA initiation, laboratory monitoring and safe stopping within nurse-led and rural community-based clinical settings.
  2.4. To evaluate the safety and tolerability of CAB-LA compared to oral PrEP.

## Methods

### Trial design

LAPIS is a phase 3B, hybrid type 1a effectiveness and implementation, parallel-group, two-arm cluster randomised controlled trial (cRCT) designed to evaluate the initial scale-up phase of offering CAB-LA, DapiRing, and PEP as additional options to oral PrEP for HIV prevention in community-based integrated HIV and SRH services. This is a hybrid design type 1a as the primary aim is effectiveness and the secondary aim is to better understand implementation outcomes. This study will be conducted, analysed and reported according to the Consolidation Standards of Reporting Trials (CONSORT) statement for cluster RCTs [20, 21].

**Fig. 1:**
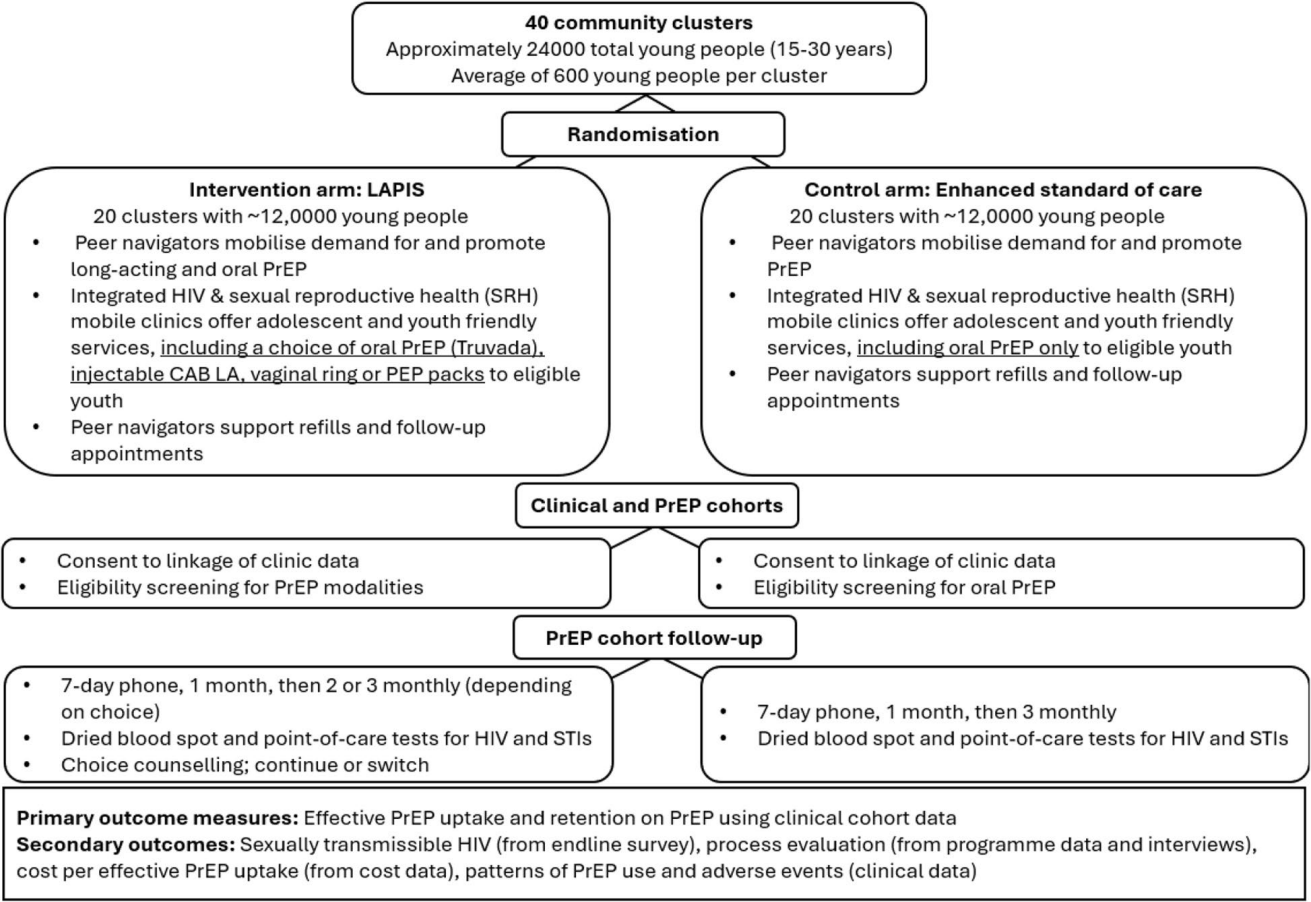
Summary of the trial design

LAPIS is embedded within the ongoing stepped-wedge trial, *Thetha nami*, which has two periods of 24 months each and is being conducted in the same 40 clusters (administrative areas). The design of the *Thetha nami* trial has been described in detail previously [19]. LAPIS is nested in the second period, when all clusters are receiving the *Thetha nami* intervention, a tailored psychosocial support and social mobilisation into community-based SRH and differentiated HIV prevention, including oral PrEP. As part of the *Thetha nami* intervention, each cluster has a pair of peer navigators who reside in the cluster and deliver the intervention to young people in that cluster. Each cluster is visited by a mobile nurse-led integrated HIV and SRH mobile clinic on a monthly basis. At the start of the second period, we randomly allocated the trial clusters to the additional choice of CAB-LA, DapiRing and PEP (intervention, *n* = 20 clusters) or control (*Thetha nami* alone, *n* = 20 clusters). The overall trial design is summarised in Fig. 1.

### Study setting

This trial is conducted within the Africa Health Research Institute’s (AHRI) health and demographic surveillance site (HDSS) in uMkhanyakude district in rural KZN, South Africa [22]. The HDSS includes a multidisciplinary adolescent and youth program that employs community-based participatory research to develop, implement, and evaluate HIV prevention interventions [23–28]. The surveillance area, with a population of 24,000 AYA aged 15–30 years, faces over 85% youth unemployment and a high burden of HIV. The area has 11 PHC that have been offering oral PrEP since 2021, but uptake remains low due to limited primary care service use among young people [29].

AHRI also implements the AHRILink health information system across the 11 clinics in the HDSS, where an AHRI clinical research associate (CRA) at each clinic records the date and reason for attendance for all consenting individuals, linking them to their unique HDSS identification number during their visit. Using this unique identifier, we can link consenting young people who engage with peer navigators or study clinics to information collected in AHRI’s HDSS surveys, and with cross-sectional surveys conducted as part of the *Thetha nami* trial. This integrated data collection allows us to measure the population-wide reach and coverage of effective HIV prevention and outcomes among all consenting individuals aged 15–30 residing in the area.

### Study population

LAPIS is being conducted in 40 clusters that have been purposively selected from the HDSS for the *Thetha nami* stepped-wedge trial, to reflect a range of rural, peri-urban and urban settings. The clusters have distinct boundaries of roads and rivers to minimise the risk of contamination/spill-over between the clusters. Since the trial is being conducted in a well-characterised HDSS, we have detailed understanding of the sociodemographic composition of the areas, as well as the numbers and population density of the young adult population.

### Eligibility criteria

At the start of the second period of the parent stepped wedge trial, all 24,000 young people aged 15–30 years residing in the 40 geographical areas (clusters) of the uMkhanyakude district in rural KwaZulu Natal will have access to the *Thetha nami* intervention. As this is a cRCT of real-world implementation, we will offer PrEP throughout the study period. Therefore, all young men and women aged 15–30 who are residing in the 40 administrative clusters in the uMkhanyakude district during the second period of the cRCT are eligible for inclusion in LAPIS.

The effectiveness outcome of uptake will be based on clinical data subject to consent. We define the ‘clinical cohort’ as all eligible 15–30-year-olds who are resident in the HDSS and attend any integrated HIV/SRH service, throughout the second step of the trial period and provide informed consent for their clinical data to be used for information on uptake of PrEP and other biomedical HIV prevention interventions. Inclusion criteria for clinical cohorts is as follows:

- Age 15–30 residing in the 40 trial clusters (control and intervention arms)
- Attend any integrated HIV/SRH service during the trial period
- Provide informed consent for their clinical data to be used for research
- Both men and women can be included

The retention outcome will be measured in a sub group of the clinical cohort who are HIV negative and sexually active and consent to being offered PrEP as part of LAPIS within the clinical cohort (PrEP cohort): All HIV negative sexually active young people aged 18–30 who attend the study research clinics will be asked to provide written informed consent for the PrEP cohort, including calling them for an exit interview at the end of the study and using their clinical data for uptake and retention on PrEP and other biomedical HIV prevention interventions. Those who are aged 15–17 will be asked to provide assent and are asked for a parent/guardian contact details to provide informed consent for an exit interview at the end of the study and clinical data usage.

There are three types of antiretroviral-based HIV prevention options that participants who test negative for HIV can opt for: 1) delivery of oral PrEP [tenofovir disoproxil fumarate and emtricitabine (TDF/FTC)] or PEP [lamivudine and dolutegravir (TLD)]; 2) delivery of injectable CAB-LA; 3) delivery of DapiRing.

#### Inclusion criteria for offering oral PrEP or PEP-in-pocket:

- Age 15–30 residing in the 40 trial clusters (control and intervention arms)
- Has provided informed consent (aged 18 and above) or, informed assent and parent/guardian informed consent (if aged 15–17)
- Documented HIV negative test
- Suitable for PrEP according to South African guidelines [30] and/or already on oral PrEP
- Understand the required dosing schedule and HIV testing
- Aware that their details can be shared with a peer navigator to support their follow-up HIV testing
- Has no history or presence of allergy to the study drugs or their components

#### Additional inclusion criteria for offering injectable CAB-LA:

- Age 16–30 residing in one of the 20 clusters randomised to the intervention arm
- Weight > 35 kg
- If pregnant or breast feeding and/or planning to become pregnant and understands that safety in pregnancy or breast feeding has not been established, but is at high risk of acquiring HIV and will not be able to take oral PrEP
- Understand the required dosing schedule and HIV testing
- Not taking any medication that is contraindicated (Carbamazepine, oxcarbazepine, phenobarbital, phenytoin, Rifampin, rifapentine)
- Has no severe mental health disorder

#### Additional inclusion criteria for offering DapiRing:

- Age 18–30 years residing in one of the 20 clusters randomised to the intervention arm
- Not pregnant

### Study interventions

Clusters have been randomly allocated to the enhanced SoC (*Thetha nami ngithethe nawe)* or enhanced SoC with the choice of injectable PrEP, DapiRing and PEP-in-pocket (intervention).

#### Control (Enhanced SoC): Thetha nami ngithethe nawe (Let’s talk)

The *Thetha nami* intervention has been described in detail previously [19]. Briefly, it is a peer-led tailored psychosocial support and social mobilisation into community-based SRH and differentiated HIV care and prevention. Area-based peer navigators mobilise youth into nurse-led mobile adolescent and youth-friendly services (AYFS) that visit fixed sites across the clusters every month. Peer navigators are men and women aged 18–30 years, selected by community leaders in each area. They are supervised by a team of peer supervisors and overseen by a committee including a professional nurse and a social worker. They participate in biweekly team debriefings and receive ongoing supervision and training.

Young people attending the mobile AYFS are offered self-taken vaginal swabs or urine tests for sexually transmitted infections (STIs), including gonorrhoea, chlamydia, trichomonas (in women), and syphilis. They also receive testing for hepatitis B and C, pregnancy testing and family planning support if female, syndromic and etiological management for STIs, HIV counselling and testing, and, if male, a referral for voluntary male medical circumcision. Those living with HIV are immediately initiated on ART, while those who test negative and are suitable according to South African National PrEP guidelines are offered oral PrEP. The initial visit is followed by a 7-day phone call and 3-monthly follow-ups. Each follow-up includes repeat HIV testing, pregnancy testing, syndromic management of STIs, and referral to peer navigators for adherence support and PrEP/ART, and contraception refills if applicable.

#### Intervention: Choice of long-acting PrEP added to Thetha nami ngithethe nawe (Let’s talk)

In addition to the enhanced SoC, the nurses in the mobile AYFS within the intervention clusters will offer the choice of long-acting injectable cabotegravir (CAB-LA) to be delivered to AYA at risk of HIV acquisition or 3-monthly DapiRings. If they choose not to take PrEP, they are offered take-home PEP-in-pocket. A PEP-in-pocket kit includes an HIV self-test, a 28-day supply of a fixed dose combination of generic Tenofovir Lamivudine and Dolutegravir (TLD), a pregnancy test and emergency contraception (for females), along with a card containing the clinic hotline and peer navigator contact information. For those who choose or switch to CAB-LA (if already on oral PrEP), the initial visit will be followed by a planned visit for the first injection. This will be followed by a one-month visit for the second injection, and then bi-monthly injections. Each visit will include repeat HIV testing, pregnancy testing, and referral to peer navigators for adherence and retention support. Safety bloods collection (full blood count, creatinine, liver function tests) will follow national guidelines.

Peer navigators in the intervention clusters promote long-acting PrEP alongside oral PrEP during community-based health promotion activities. As part of the peer mentorship package, they remind young people of their follow-up visits and if feasible, accompany those on CAB-LA to their first follow-up injection (1 month) and subsequent bi-monthly injections at the mobile clinics. With support from their supervisors and oversight committee, peer navigators trace individuals who have missed their scheduled visits. They assist clinical teams in ensuring that young people who have discontinued CAB-LA receive regular HIV testing and remain protected from acquiring HIV during the CAB-LA tail period.

### Primary outcomes

There are two primary outcomes for evaluating the effectiveness of the intervention:

1. Effective uptake of PrEP: this is defined as the proportion of young people who have taken any form of PrEP (oral, injectable, ring) or PEP-in-pocket in the study clinics. This outcome is defined based on clinical data. Individuals aged 15–30 years who are resident in the trial clusters (irrespective of HIV status) but who have not given consent to share clinical data (the vast majority of whom will not have attended a clinic so cannot have taken up PrEP) are considered not to have taken up PrEP.
2. Retention on PrEP: this is defined as attending at least one follow-up appointment after PrEP/PEP initiation, including appointments for HIV testing. Retention will be assessed among individuals who have taken any form of PrEP or PEP and have at least 4 months of follow-up by the end of the trial (referred to as the PrEP cohort).

### Secondary outcomes

Secondary outcomes of the trial include sexually transmissible HIV (HIV viral load of ≥ 400 copies per ml), uptake of risk informed HIV prevention, process evaluation (implementation) outcomes, cost per effective PrEP uptake, adverse events associated with CAB-LA compared to oral PrEP, and patterns of PrEP/PEP use. Implementation outcomes will be evaluated using the RE-AIM (Reach, Effectiveness, Adoption, Implementation, Maintenance) framework.

### Tertiary outcomes

Tertiary outcomes include self-reported contraception use and self-reported current pregnancy, sexually transmitted infections, HIV incidence, mental health and socioeconomic outcomes (educational, employment and food security).

### Sample size

Based on our current data, we estimate that 20% of young people aged 15–30 years in the enhanced SoC arm will attend a clinic to undergo an HIV risk assessment, and 20–30% will start or continue PrEP (primary outcome 1), i.e., 4%−6% (0.20 × 0.20–0.30) of all young people in the control clusters. With 12,000 AYA in the control clusters we estimate that ~ 600 will start/continue oral PrEP. Assuming intracluster correlation coefficients (ICCs) values between 0.05–0.15, with 12,000 young people in the control clusters and 12,000 in the intervention clusters, we will have 80% power to detect an increase in PrEP uptake from 4 to 9% (at ICC 0.05) or 14% (at ICC 0.15). We will have 90% power to detect an increase in uptake to 10% (ICC 0.05) or 16% (ICC 0.15) (Supplementary Fig. 1). Similarly, we will have 80% power to detect an increase in PrEP uptake from 6 to 12% (at ICC 0.05) or 17% (at ICC 0.15) (Supplementary Fig. 2).

If uptake increases to 9%–14% in the intervention arm, this will result in 1100–1700 (55–85 per cluster) young people starting PrEP or PEP over 14 months. However, our second primary outcome (retention on PrEP) is assessed only among those who have at least four months of follow-up by the end of the trial. Therefore, we have used a more conservative estimate of the numbers per cluster in our power calculations. The current retention in those who start PrEP is 20%. With a sample size of 400 (20 per cluster) in the enhanced SoC arm and 780 (39 per cluster) in the intervention arm, and assuming ICC values between 0.05 and 0.15, we will have 80% power to detect an increase in retention from 20 to 31% at an ICC of 0.05 or to 37% at an ICC of 0.15. We will have 90% power to detect an increase in retention from 20 to 33% (ICC = 0.05) or 40% (ICC = 0.15).

### Randomisation: sequence generation

The 40 trial clusters were randomly allocated in a 1:1 ratio to the LAPIS intervention or enhanced standard of care. Randomisation was stratified by early or delayed roll-out of the *Thetha nami* intervention in the first period of the stepped-wedge trial. In addition to this stratification, we applied covariate-based restricted (or constrained) randomisation to ensure that the clusters are reasonably balanced between arms with respect to key characteristics namely: population size of the 15–30-year-olds, proximity to the major road, and geographical location in the northern or southern part of the study area. Similar processes of restricted randomisation conducted in the *Thetha nami* trial [19] were followed here. The tolerance thresholds for balance were determined iteratively by testing various thresholds and evaluating the number and validity of the acceptable allocations. In evaluating our restricted randomisation scheme, we aimed to achieve a sufficient number of acceptable allocations and a reasonably uniform distribution of joint allocation probabilities. A random sample of 10,000 possible allocations of clusters to two groups (labelled A or B) was selected from a total of approximately 500,000 possible allocations that met the restriction criteria. Each allocation was assigned a unique running number from 1 to 10,000.

### Intervention assignment and concealment

On 28 September 2023, a two-stage public randomisation ceremony was conducted to ensure transparency and enhance community buy-in, following similar procedures to those detailed in the *Thetha nami* stepped-wedge cRCT trial [19]. In the first stage, a 4-digit randomisation number was drawn using numbered balls and cluster leaders, identifying the allocation sequence from a pre-generated list of 10,000 possible allocations. In the second stage, cluster leaders participated in a coin toss and card draw to determine whether group A or B would be assigned to the LAPIS intervention or enhanced standard of care.

### Blinding

Due to the nature of the intervention, blinding of participants and the intervention delivery teams is not feasible. The trial statistician independently generated the allocation sequence, and neither the statistician nor the clinical team took part in the public randomisation ceremony. Throughout the trial, investigators, statisticians, research assistants enrolling participants in surveys, and laboratory staff will remain blinded until the analysis plan is finalized, and the database is locked.

### Study visits and assessment procedures

Table 1 outlines the schedule of enrolment, interventions, and assessments for the LAPIS trial, detailing the timing of key activities across both intervention and standard-of-care clusters.

**Table 1:** Schedule of enrolment, interventions, and assessments for LAPIS cluster randomised trial

	**Study period**		
	**Allocation (clusters)**	**Intervention delivery**	**Endline survey**
Timepoint	***Month 0***	***Month 1–15***	***Month 16–24***
Enrolment:			
Eligibility screen		X	X
Informed consent		X	X
Restricted randomisation of *N* = 40 clusters	X		
Allocation	X		
Interventions:			
*Choice of long-acting injectable PrEP with cabotegravir, DapiRing or post-exposure prophylaxis-in-pocket (PEP-in-pocket) within community based integrated HIV (PrEP/ART) and SRH services*		20 clusters	X
*Enhanced standard of care: Peer-led mobilisation into community based integrated HIV (oral PrEP or ART) and SRH services*		20 clusters	X
Assessments:			
*Blood sample for HIV testing & viral load if positive*			X
*Urine and self-taken vaginal swabs for STI testing*			X
*20–30-min interview- administered questionnaire (uptake of HIV & SRH services, sexual risk, reproductive health, mental health *etc*.)*			X
*Clinical data from clinical management tools*		X	X
*Programme data from peer navigators’ support management tools*		X	X
*In-depth interviews with peer navigators, clinic staff and young people*		X	X
*Costing Data*		X	X

### Clinical procedures for the enhanced SoC arm

The study mobile clinics offer nurse-led HIV testing, prevention, and care, integrated with SRH services. These services include adolescent and youth-friendly, gender-neutral, and HIV status-neutral individualised risk assessments for HIV care and PrEP, catering to key populations. During the SRH clinic appointments, participants receive counselling on sexual health, fertility intentions, contraception, emergency contraception, and HIV counselling and testing. All clinic attendees are offered pregnancy testing, family planning support, choice of contraception, syndromic management for STIs and if male referral to voluntary male medical circumcision. Everyone is offered HIV counselling and POCT, and immediate initiation of ART if found to be living with HIV. This approach integrates sexual health counselling with PrEP to maintain negative HIV status and ART to ensure wellness and viral suppression (undetectable = untransmittable).

All those who are HIV negative are counselled for PrEP or post exposure prevention (PEP) suitability according to South African National guidelines. HIV testing will follow South African national Department of Health guidelines, involving two POCTs and a third test if results are discrepant. A dry blood spot (DBS) or venous blood sample will be sent for HIV ELISA testing at our accredited diagnostic laboratory, confirming HIV-negative status with a 4th generation ELISA test including the p24 HIV antigen. Positive HIV tests will lead to viral load and resistance testing, with further ELISA testing for discrepancies. Those who are enrolled to the study are also offered testing for sexually transmitted infections, including POCT for syphilis and hepatitis B surface antigen, hepatitis C antigen, self-taken vaginal swabs or urine tests for gonorrhoea and chlamydia (and trichomonas in women), and treatment and partner notification if positive. Blood samples will also be sent to the National Health Laboratory Service for creatinine and liver function tests.

If a participant agrees to immediate PrEP or ART initiation, they are provided with a 28-day supply of tenofovir disoproxil fumarate and emtricitabine (TDF/FTC) for PrEP, or tenofovir disoproxil fumarate, emtricitabine, and dolutegravir (TLD) for ART. Participants receive a phone call seven days after initiation to receive the results of their STI testing and blood tests and complete a standard symptom screen for adverse effects and are referred to a clinic for care if needed. A mobile clinic appointment is scheduled one month after PrEP/ART initiation. According to national guidelines, mobile clinic appointments for refills and monitoring are scheduled every three months thereafter. We also provide community refills aiming for continuous PrEP supplies. The complete schedule for visits is outlined in Supplementary Table 1.

### Clinical procedures in the intervention arm

In addition to the procedures above, all those who are HIV negative are counselled for PrEP or PEP choice using the South African department of health job aids. This will include a choice of long-acting injectable CAB-LA, oral PrEP, DapiRing, or PEP-in-the-pocket. Individuals will receive information on long-acting injectable CAB-LA, oral PrEP, DapiRing, or PEP-in-the-pocket efficacy, potential side effects, the follow-up schedule, and adherence importance.

Participants will either receive their first Apretude (600 mg/3 ml) intragluteal injection administered by a professional nurse, or a 28-day supply of TDF/FTC for PrEP or a DapiRing (vaginal insertion) or PEP pack. Participants receive a phone call seven days after initiation to receive the results of their STI testing and blood tests and complete a standard symptom screen for adverse effects and are referred to a clinic for care if needed. They will have access to a clinical hotline for follow-up appointments and community-based HIV testing if treatment is discontinued.

The mobile clinic appointments for refills and monitoring will be at month one (M1) for all participants and then bi-monthly for CAB-LA and three-monthly for oral PrEP, DapiRing, or PEP-in-pocket. Participants will undergo HIV and pregnancy POCTs and DBS for HIV ELISA and antigen testing at each follow-up (see Supplementary Table 1 for the complete schedule). Those not opting for CAB-LA, DapiRing or oral PrEP will be offered PEP-in-pocket for use after condomless sex. All participants will be offered a clinical hotline and a peer navigator for support to help them attend follow-up appointments and arrange HIV testing in the community once/if they discontinue.

#### Pregnancy and breastfeeding

Women will be informed that the safety of CAB-LA during pregnancy and breastfeeding is not established, and that CAB-LA may remain in the body and breast milk for up to 12 months after discontinuation. They will also be offered long-acting contraception. Although the South African Health Products Regulatory Authority (SAHPRA) approval does not contraindicate CAB-LA use during pregnancy and breastfeeding, the decision to start or continue CAB-LA for high-risk women will be based on an individual risk–benefit discussion. The study doctor will consider CAB-LA use on a case-by-case basis if the known risk of HIV acquisition outweighs the unknown risks of CAB-LA, and if the participant fully understands the lack of established safety and is unable or unwilling to use oral PrEP or other prevention methods. Enhanced safety monitoring will be conducted both antenatally and postnatally.

### Criteria for discontinuing or modifying allocated interventions

Nurses and peer navigators will continue to engage participants, tailoring HIV prevention strategies to their risk levels, including stopping and starting PrEP. Participants experiencing adverse effects will be referred to a fixed clinic for care if needed. If a participant seroconverts while on PrEP (indicated by a new positive ELISA test), ART will be initiated immediately, HIV resistance will be tested, and HIV viral loads will be monitored. We will report every case of pregnancy among the PrEP cohort to the Trial Steering Committee (TSC)/Data and Safety Monitoring Board (DSMB), ViiV Healthcare, the South African National Department of Health (NDoH), and the Antiretroviral Pregnancy Registry (APR) within one week of becoming aware of the pregnancy, including the pregnancy outcome.

#### Switching between oral and injectable PrEP

This is an implementation study assessing the real-world use of CAB-LA as a choice alongside oral PrEP and so participants will be allowed to switch. Participants will be counselled on the importance of HIV testing and additional HIV protection, including oral PrEP and condoms, to reduce the risk of HIV acquisition and resistance during the 12 months following the discontinuation of CAB-LA.

#### Adverse events and laboratory monitoring

As one of the earliest real-world uses of CAB-LA, we will adhere to the national guidelines released in November 2023, and follow the clinical procedures outlined above. Participants will start on PrEP (oral or CAB-LA, depending on trial arm and participant choice) based on POCTs conducted on the day of their visit to confirm eligibility (HIV negative status and not pregnant). We will monitor full blood count (FBC), creatinine (for estimated glomerular filtration rate calculation—eGFR), and liver function tests at screening and exit from study, and as clinically indicated. All participants will be informed about potential side effects and adverse events and will receive a clinical hotline and peer navigator contact details for reporting concerns. In the event of hepatotoxicity (more than twice the upper limit of normal), renal toxicity (eGFR < 60 mL/min or a sustained 25% decrease in eGFR from baseline), hypersensitivity, new depression, or suicidal behaviours, the participant will be seen by the study doctor for further investigation. This may include discontinuation of PrEP, management of the adverse event, or referral to specialist services. The study doctor will continue to follow up with the participant until the adverse event is resolved.

Hepatitis B and C: Those who are hepatitis B surface antigen negative and who have not been vaccinated are offered hepatitis B vaccination. In the uncommon situation where an individual tests positive for hepatitis B or C antigen, they undergo further hepatitis virus testing, full blood count, liver function tests, liver ultrasound and other recommended imaging. They will be reviewed by our study doctor for management according to the South African guidelines and onward referrals to specialist services. In the event that they have chronic hepatitis B and are eligible to initiate PrEP with tenofovir disoproxil fumarate and emtricitabine (TDF/FTC) or ART with tenofovir disoproxil fumarate, emtricitabine and dolutegravir (TLD) they are counselled that tenofovir disoproxil fumarate, emtricitabine also treats the Hepatitis B virus and that clinical trial and marketed use of emtricitabine and tenofovir has shown that some participants with chronic Hepatitis B virus disease may experience clinical or laboratory evidence of recurrent hepatitis upon discontinuation of these treatments. We therefore counsel them against unplanned discontinuations or switching of the emtricitabine or tenofovir and strongly recommend that in the event of discontinuation to attend our services for monitoring of liver chemistry tests and markers of hepatitis B virus replication.

### Strategies to improve adherence and retention

Participants on PrEP are offered HIV testing every two to three months (depending on whether they are on injectable or oral PrEP) and annual STI testing. Those on ART receive annual HIV viral load assessments. All individuals starting PrEP, ART, or contraception are provided with peer navigator support as part of their personalized adherence plan, including assistance with refills, appointment scheduling, and reminders. We will maintain our robust follow-up system currently used for oral PrEP, which includes phone calls, peer navigators, and research assistants tracking participants, addressing missed appointments, and understanding the reasons behind them. Neutral text message reminders are sent to participants who have access to private messaging, and a clinical hotline is available for contact at any time.

### Outcome ascertainment

In Table 2 we define the primary and secondary outcomes in more detail and provide details of the data sources for each outcome. Data for the primary outcomes will be collected from the clinical cohorts comprising young people aged 15–30 years residing in the HDSS who attend the study mobile clinics in the surveillance area. Secondary outcomes and implementation outcomes will also utilise data from other sources: i) cross-sectional population-based surveys at baseline and endline of a random sample of 3200 young people aged 16–30 years residing in the trial clusters; ii) programme data from approximately 24,000 individuals aged 15–30 years eligible for intervention; iii) qualitative data collected during the process evaluation; vi) costing data.

**Table 2:** Summary of the primary and secondary outcomes in LAPIS trial

Outcome	Definition	Data source
Primary outcome		
1. Effective uptake of PrEP or PEP	Uptake is defined as the proportion of young people who have taken any form of PrEP (oral, injectable, ring) or PEP-in-pocket in the study clinics. This outcome will be assessed among all individuals aged 15–30 years who are resident in the trial clusters irrespective of HIV status	Clinical cohorts
2. Retention on PrEP	Retention is defined as attending at least one follow-up appointment after PrEP/PEP initiation, including appointments for HIV testing. Retention will be assessed among individuals who have taken any form of PrEP or PEP and have at least 4 months of follow-up by the end of the trial	PrEP cohorts
Secondary outcome		
1. Prevalence of sexually transmissible HIV	We use a composite outcome that captures the effect of the intervention on both incident HIV and untreated HIV. We will measure the prevalence of sexually transmissible HIV as the proportion of those living with HIV and have a detectable HIV viral load, defined as having an HIV viral load of ≥ 400 copies/mL, during our endline survey round	Endline survey
2. Intervention uptake	We will measure the uptake of universal risk informed HIV prevention intervention as a composite outcome defined as the proportion of participants who are aware of their HIV status and are either on ART if living with HIV or have ever taken up PrEP following a risk assessment if HIV negative. This outcome will be defined based on laboratory data and linkage to clinical data (AHRI Link and mobile clinical data through HDSS number)	Baseline and endline surveys and clinical cohort data
3. HIV acquisition or transmission	Proportion of men and women aged 16–30 at risk of acquiring HIV or transmitting HIV. This will be measured as a composite outcome to represent risk of acquisition or transmission defined as engaging in condomless sex in the last 3 months and either HIV negative and not taking PrEP during that period or living with HIV and not virologically suppressed (VL > 400 copies/ml)	Baseline and endline surveys and clinical cohort data
4. Process evaluation (implementation)	RE-AIM (Reach, Effectiveness, Adoption, Implementation, Maintenance) framework fully defined in Table 3	Table 3
5. Cost per effective PrEP uptake,	Cost per effective PrEP uptake (Maintenance): The primary implementation outcome is cost-effectiveness (cost per case linked to effective PrEP). We will measure the costs in the intervention and control arm, to compare the two arms in their cost-effectiveness in achieving endpoints, i.e., the cost per case linked to effective PrEP	Cost data
6. Adverse events	Adverse events with CAB LA compared to oral PrEP. Proportion discontinue or switch due to adverse events in each arm	Clinical cohorts
7. Patterns of PrEP/PEP use	We will describe the patterns (i.e. continuation or discontinuation) of PrEP/PEP by the intervention arm	Clinical cohorts

### Clinical and PrEP cohorts

All young people aged 18–30 years who attend either PHC clinics in the HDSS or the study research clinics will be asked by the AHRI CRAs to provide informed consent for their clinical data to be used for information on uptake of PrEP and other biomedical HIV prevention interventions (clinical cohorts). Those who are HIV negative, sexually active and attend the study clinics during the trial period will be invited by the study nurse to enrol into the PrEP cohorts. Those aged 15–17 year will be asked to provide assent and to provide a parent or guardian’s contact to give informed consent for data use. For participants at the study clinics who consent to data usage, relevant clinical data will be extracted from the clinical management tool. These data will include HIV testing, ART uptake, PrEP eligibility screening, PrEP (oral, injectable, or ring) uptake, and other services (such as PEP or PEP-in-pocket received). We will also measure retention, adherence, and reasons for stopping or restarting PrEP in the PrEP cohorts. At the 11 clinics in the surveillance area, the AHRILink system will be used to identify individuals from the study area who attend PHC clinics for oral PrEP.

### Cross sectional surveys

We will use the AHRI HDSS as a sampling frame to randomly select a sample of 6,400 individuals (160 per cluster) aged 16–30 years at baseline and at endline. We are using the same surveys as described previously in the *Thetha nami* stepped-wedge cRCT [19]. Sampling will be stratified by sex and age group. Based on pilot trial experience, we expect approximately 4,800 individuals to be contactable and eligible, and 3,200 (80 per cluster) likely to provide consent. Research assistants will visit sampled individuals at home to provide study information and invite them to participate. Interested individuals will receive a chance to ask questions and, if aged 18 or older, provide written informed consent. Those aged 16–17 will be asked to provide assent, with written parental consent.

After consent/assent, participants will complete a 20–30-min interviewer-administered questionnaire on an electronic tablet, with sensitive questions being self-completed. The questionnaire will cover:

- Awareness and uptake of HIV status, oral and injectable PrEP, ART, contraception, and wider HIV prevention measures.
- Sociodemographic information.
- Sexual risk behaviours (e.g., number of partners, condom use, transactional sex).
- Reproductive health (e.g., contraception, pregnancy, fatherhood, voluntary medical male circumcision (VMMC)).
- Mental health (using PHQ9, alcohol and drug use, and violence assessments).

Participants will be offered HIV POCT and will provide DBS and/or paediatric blood collection tubes (< 2 ml) for HIV ELISA and viral load testing if they test positive. We will ask for consent to link their survey data with clinical data collected in the mobile AYFS and programmatic data from peer navigators and primary health care settings through the AHRILink system.

### Programme data

Patterns of programme uptake and retention will be measured in aggregate among all young people aged 15–30 years who reside in the 40 administrative clusters. These data will be aggregated by age and sex only.

### Process evaluation

To better understand community-based implementation of CAB LA in this rural context, we will a conduct a mixed method process evaluation. We will measure implementation outcomes using the RE-AIM (Reach, Effectiveness, Adoption, Implementation, Maintenance) Framework (Table 3).

**Table 3:** RE-AIM process evaluation and Implementation outcomes

Dimension	Implementation outcome	Data source	Obj
Reach	Client (user) level: The extent to which key populations and those at highest risk of HIV acquisition uptake PrEP, and what are the patterns of uptake and retention. We will measure this quantitatively as the proportions of the target population who are aware and uptake PrEP from our endline survey as well as the differences in the patterns of uptake and retention of different PrEP/PEP per arm with a focus on the hard-to-reach groups (out of school, recently migrated, living in remote areas and key populations)	Endline survey of the trial & programme data, clinical cohorts	2.1
Effectiveness	Client (user) level: Increased effective use (adopt and adhere) to PrEP amongst 15–30-year-olds in choice versus SoC arm. This is measured as proportion who undergo risk assessment, uptake PrEP and are retained on PrEP	Clinical cohorts (cRCT)	1
Adoption	Client (user) level: Acceptability and appropriateness (attitudes towards and PrEP preferences and HIV testing preferences) amongst 15–30-year-olds. These are dynamic characteristic and will be assessed, both through the surveys and in-depth qualitative interviews with participants	Qualitative interviews with service users and endline survey of clinical cohort	2.1
Implementation	Provider (service) level: Feasibility, and fidelity of adding a choice of CAB LA in the intervention arm. We will capture how CAB LA is delivered in practice with a focus on specific implementation questions including use of HIV self-testing for the three stages or initiation, continuation, and safe stopping	Programme data, & Qualitative interviews and debriefings with service providers	2.2 and 2.3
Maintenance	Provider and Health Systems level: Affordability, and resources needed for scalability through primary care and community settings	Costing & moderated discussion with stakeholders	2.2

We will conduct serial in-depth interviews with 50 participants, purposively selected to ensure a diverse representation of sex, age, geographic area, and exposure to intervention and control arms. Additionally, we will interview six study nurses or CRAs and ten peer navigators. Natural group discussions will be conducted with seven community groups and intervention delivery staff: one with clinic staff, two with peer navigators, and four with community members. Peer navigators and clinical staff will be trained to maintain brief daily notes on their activities. These notes will facilitate reflection during debriefing sessions, ongoing training, and supervision. Drawing on our extensive experience in participatory ethnographic research, we will ensure that staff are trained in proper note-taking procedures, emphasizing the exclusion of personal or identifying details such as dates, specific meeting locations, or memorable anecdotes.

### Costing data

We will collaborate with HE^2^RO (Health Economics and Epidemiology Research Office, University of Witwatersrand) our partner in conducting the health economics analysis for the parent trial, to establish costs. We will utilize data collection tools developed by HE^2^RO alongside those used in previous trials to measure intervention costs and gather bottom-up ingredient-based costs. Additionally, we will employ a top-down costing approach using AHRI study budgets and expenditure reports. To capture out-of-pocket costs, we will include patient cost cohorts, which will be added to the intervention costs.

### Data management

Data collectors will administer surveys using tablets to ensure real-time data capture. The questionnaire will be programmed using the REDCap software [31] and accessed via the tablet application. Automatic checks will be programmed in REDCap to check for invalid values, internal inconsistencies, and implausible responses, with additional validation checks conducted after data collection. Data from REDCap will be uploaded to a MySQL database server within a secure server cluster at AHRI. Laboratory data will be electronically output from the Laboratory Information Management System (LIMS) and imported into the database. Qualitative data will be stored in Word files or Excel spreadsheets and can be uploaded into NVivo for management and analysis. These files will be kept in a secure, access-controlled folder on an AHRI file server. All study-specific data will be stored on an AHRI server with strictly controlled access.

### Statistical analysis

Analysis of the two co-primary outcomes (uptake of PrEP, and retention on PrEP among those who initiate) will be based on fitting random effects logistic regression models with random effects for each cluster to estimate the odds ratios (OR) and 95% confidence intervals (CI), adjusting for the design factors (exposure during the first period of *Thetha nami*, and factors used in the restricted randomisation). To handle any chance imbalance in the distribution of important covariates between arms, and potentially gain power, we will also adjust for pre-specified cluster- and individual-level factors that may be associated with PrEP uptake in the population.

The analysis of retention will be restricted to individuals who initiate PrEP, in any of the clinics in the AHRI HDSS or the mobile AYFS during the trial period (i.e. the PrEP cohort).

We will not make any adjustments for multiplicity in our analyses, despite having two primary outcomes, because we believe these outcomes correspond to distinct research questions and because we will not declare the intervention successful based simply on a significant benefit observed for one outcome (but rather will consider evidence from all outcomes holistically). A detailed analysis plan will be developed and finalised before database lock.

### Harms

This is a pragmatic trial, with all tests and drugs already approved by the South African Health Products Regulatory Authority (SAHPRA) for clinical use in South Africa. Clinical care will adhere to South African department of health clinical guidelines, and the risk of harm is anticipated to be low. Adverse Events (AEs) and Serious Adverse Events (SAEs) will be captured through clinics, process evaluation, community engagement units, community advisory boards, the hotline, as well as peer navigators and clinic staff. These will be logged using a Clinical Reporting Form (CRF) and SAE CRF for up to 12 months after the intervention begins. Reported AEs and SAEs will be monitored, categorized using an established grading system, and followed up by AHRI. The Standard Level of SAEs will be used for reporting to study leadership within two working days of awareness: all deaths, disabilities/incapacities, hospitalizations that are suspected adverse drug [procedure] reactions (where a relationship to study procedures cannot be ruled out), congenital abnormalities and birth defects, and all other Grade 4 events that are suspected adverse drug [procedure] reactions.

SAEs will be logged, with the Principal Investigator evaluating the SAE for seriousness and likely relationship to the intervention. Related SAEs will be reported to UKZN Ethics Review Boards, ViiV and trial steering committee within 24 h of becoming aware of the event. All AEs and SAEs will also be reported though quarterly progress reports to TSC/DSMB meetings. Annual reports with full listings of SAEs will also be submitted to Ethics Review Boards.

### Trial oversight

A TSC/DSMB has been assembled and will meet twice a year, or more frequently, if necessary, to ensure the study is conducted with rigor and to provide advice to the Principal Investigators (PIs), research team, and implementing partners. The DSMB comprises independent international and local experts in scientific research. It is responsible for overseeing trial progress against the protocol, ensuring respondent safety, and considering any new information relevant to the research objectives. In addition, a Project Management Group (PMG) has been established, comprising the PI, study coordinators, statisticians, data managers, social scientists, and team leaders (research assistants). The PMG holds weekly meetings to review data monitoring reports and address study needs such as screening, recruitment, enrolment issues, and medication adjustments for participants. The project coordinators and team leaders manage the day-to-day operations of the study. Matters requiring further deliberation are escalated to the Co-Investigators and, if necessary, to the DSMB for resolution.

### Provisions for post-trial care

During the planning phase, we collaborated with the National and Provincial Department of Health to ensure our interventions would generate data needed for scaling up. We established a working group of health officials leading HIV testing, prevention, and SRH in the district, along with a technical advisory group in KZN province. These groups helped us optimize and integrate our community-based PrEP delivery with primary health care, determine the location and distribution of mobile clinics, and develop the evaluation framework.

After the trial, all study participants will continue to receive ART or oral PrEP and contraception through public health facilities. To ensure a smooth transition, AHRI’s AHRILink software will flag study participants to AHRI CRAs at the reception of the 11 PHC clinics and/or our district hospital. Participants will be directed to the AHRI nurse, who will provide the first three months of care and facilitate the transition to the public health system standard of care for ART or oral PrEP.

Although CAB-LA is currently approved by SAHPRA, it is not yet available through public health facilities in our district. However, we are part of the South African National Department of Health long-acting PrEP implementation group. Through this forum, implementation experiences from LAPIS and other studies will contribute to the development of South African long-acting PrEP national guidelines. If CAB-LA is included in the national PrEP roll-out, we anticipate that publicly funded health care facilities in our setting will be included in the early stages. Our study participants will then have the option to continue with or switch to CAB-LA, either through our HIV prevention services or by transitioning to public health facilities.

### Ethical considerations

All procedures contributing to this study comply with the ethical guidelines and standards of the relevant national and institutional committees on human experimentation and with the Declaration of Helsinki. Ethical approval has been obtained from the University of KwaZulu-Natal Biomedical Research Ethics Committee (BREC/00003735/2021) and UCL Research Ethics Committee (5672/006). For any additional data collection required for the costing, ethical approval has been obtained from the University of Witwatersrand Human Research Ethics Committee (Medical) (HREC 220708)) and Boston University IRB (H43001).

We will clearly distinguish between research activities and service delivery. Research components will include data for outcome evaluations from cross-sectional surveys, qualitative interviews, group discussions and clinical data amongst those who attend the AYFS. Written informed consent will be required for all individuals aged 18 and over, while those aged 15–17 will require parental or guardian consent and their own assent to participate in research. In contrast, the SRH and HIV care services will be considered service delivery rather than research. Consent in this context pertains to receiving healthcare services and will adhere to the guidance provided by the South African National Department of Health. As CABLA, DapiRing, and PEP-in-pocket are not current standard of care PrEP in South Africa, all clinic attendees who are eligible and would benefit from PrEP will also undergo a process of informed consent in those aged 18 and over, and informed consent from parents and legal guardians and informed assent for participation in research in those aged 16 to 17. All staff (including peer navigators) are provided with training on research ethics such as confidentiality, voluntary participation and good clinical practices. All participants have the right to withdraw from the study at any time. It will be clearly communicated to participants that their decision to refuse participation or withdraw from the study will not impact their access to health-related services, including peer navigator support and clinical services provided at the study mobile clinics and PHC clinics.

Participants in the cross-sectional surveys will receive compensation or airtime equivalent to 150ZAR and reimbursement for any travel costs incurred. In accordance with SAHPRA requirements, participants who have received at least one dose of the investigational products (CAB-LA, DapiRing, PEP-in-pocket, or oral PrEP) through the study clinics will receive 400ZAR for completing a longer survey and laboratory testing. As this is a real-world implementation study, individuals receiving clinical services, including PrEP, will not receive any incentives or reimbursements.

### Plans for communicating important protocol amendments

Any trial design changes, including modifications to eligibility criteria, will be reported to the DSMB for guidance and submitted for review by the relevant Ethics Committee. Upon approval, the changes will be updated on ClinicalTrials.gov.

### Dissemination plans

The results of the trial, protocols, study tools, data analysis plans, and anonymized data sets will be uploaded to ClinicalTrials.gov, published in open access peer-reviewed journal and data repositories and presented at national and international conferences.

## Discussion

LAPIS is one of the first pragmatic trials to evaluate the population effect of integrating long-acting injectable PrEP and DapiRing as additional PrEP modalities to oral PrEP within community-based sexual and reproductive health services for adolescent and young adults at substantial risk of HIV. Stakeholders, including young people, community members, and health officials, have emphasised the urgent need for diverse PrEP options, particularly long-acting injectable PrEP, in rural and poor areas of southern and eastern Africa [32–35]. These regions face a high burden of HIV and poor sexual health outcomes.

We have actively engaged young people from the inception of our PrEP programmes. Extensive community-based participatory research was conducted to iteratively co-create the peer navigator component, ensuring that the interventions are tailored to the needs and preferences of young people [27]. We continue to engage youth throughout the study including, but not limited to, the youth advisory component of our community advisory board and delivery of interventions through peer-navigators. This engagement ensures that the study remains responsive to the needs and preferences of young people.

To address the critical needs identified by stakeholders, the study focuses on several key implementation goals. First, it aims to understand the real-world implementation of CAB-LA by evaluating the end-to-end community model of CAB-LA delivery to ensure its feasibility and effectiveness in real-world settings. Second, the study seeks to implement CAB-LA through nurse-led mobile and fixed facilities that align with the existing models of rural healthcare delivery in South Africa, particularly those used by the National Department of Health and KwaZulu-Natal provincial health services. Third, the trial aims to measure the impact of choice on uptake and retention at a population, and not facility or service level, providing evidence of reach and coverage of community-based delivery of choice in rural settings. Finally, the study emphasises embedding universality, gender-neutrality, and integration with SRH to reduce stigma and ensure broad accessibility. This approach is designed to also reach hidden key populations, such as young women who sell sex and AYA who may not be aware of their risk of HIV acquisition.

LAPIS offers a person-centred approach to HIV prevention that includes a choice of PrEP modalities enabling young people to have more options to suit their needs, thereby improving adherence and retention. Through extensive stakeholder engagement and a focus on practical implementation strategies, we seek to provide scalable and effective solutions to reduce HIV incidence and improve sexual health outcomes in high-burden settings.

### Trial status

The version of this protocol is 8.0 (dated 05 November 2024). Screening and enrolment to the LAPIS trial began on 27 February 2024 and is anticipated to continue until the end of August 2025.

## Supplementary Information

Supplementary Material 1.

## Data Availability

No datasets were generated or analysed during the current study.

## Abbreviations

AGYW: Adolescent Girls and Young Women
AHRI: Africa Health Research Institute
ART: Antiretroviral therapy
DSMB: Data Safety And Monitoring Board
HIV: Human Immunodeficiency Virus
KZN: KwaZulu-Natal
PHC: Primary Health Care Clinic
POCT: Point of Care Test
PrEP: Pre-Exposure Prophylaxis
SA: South Africa
SAE: Serious Adverse Events
SOC: Standard of Care
SRH: Sexual Reproductive Health
STI: Sexually Transmitted Infection
SWT: Stepped-wedge trial
TDF/FTC: Tenofovir disoproxil fumarate and emtricitabine
TSC: Trial Steering Committee
UCL: University College London
UKZN: University of KwaZulu‑Natal
UTT: Universal Test and Treat
