# Supplementary material for: Long-acting HIV pre-exposure prophylaxis integrated with community-based sexual and reproductive health services in South Africa (LAPIS): study protocol for a hybrid (1a) cluster randomised controlled phase 3B trial of effectiveness and implementation
Source: BMC Public Health. 2026 Jan 20;26:610. doi: 10.1186/s12889-025-24889-1 (PMC12908261; doi:10.1186/s12889-025-24889-1)
Supplement: Supplementary file 1 — Supplementary Material 1. [file 12889_2025_24889_MOESM1_ESM.docx]

Supplementary Table 1 Schedule visits

|  | **TDF/FTC or DapiRing or PEP-in-pocket** | **CAB-LA** |
| --- | --- | --- |
| **Screening (clinical research assistant)** | HIV point of care testing x2. If HIV negative and weigh over 35 kg offered participation in study. | HIV point of care testing x2. If HIV negative and weigh over 35 kg offered participation in study. |
| **Day 0 (clinic nurse)** | HIV negative and eligible for and consent to PrEP, taken venous blood for 4^th^ generation HIV antibody/antigen test, hepatitis B surface antigen, hepatitis C antigen, syphilis, Full Blood Count (FBC), creatinine and liver Function Tests (LFT). Urine for pregnancy test. Sexually transmitted infections, including self-taken vaginal swabs or urine tests for gonorrhoea and chlamydia. Offer contraception  Dispense 28-day supply of tenofovir disoproxil fumarate/emtricitabine (TDF/FTC)  Refer to Peer Navigator for support or DapiRing or PEP-in-pocket | HIV negative, eligible for and consent to PrEP and choose CAB-LA. take venous blood for 4^th^ generation HIV antibody/antigen test, hepatitis B surface antigen, hepatitis C antigen, syphilis, Full Blood Count (FBC), creatinine and liver Function Tests (LFT). Urine for pregnancy test. Sexually transmitted infections, including self-taken vaginal swabs or urine tests for gonorrhoea and chlamydia. Offer contraception  Receive x1 intragluteal injection for Apretude (600mg/ 3ml)  Liaise with peer navigator for follow-up |
| **Day 7** | Phone appointment for symptoms, adherence and to review STI and HIV Elisa, Hep B and C tests, creatinine, FBC and LFTs | Phone appointment to review STI and HIV Elisa, Hep B and C tests, creatinine, FBC and LFTs |
| **Month 1 (M1)** | HIV point of care testing x2 and dry blood spots for 4^th^ generation HIV antibody/antigen test, pregnancy test.  Contraception if needed and check happy / need to continue and or desire to switch (in choice arm)  Adherence support and liaise with peer navigator  Dispense 3-month supply of tenofovir disoproxil fumarate and emtricitabine (TDF/FTC) or DapiRing or 1XPEP-in-pocket | HIV point of care testing x2 and dry blood spots for 4^th^ generation HIV antibody/antigen test, pregnancy test, FBC, LFT, and creatinine.  Contraception if needed and check happy / need to continue and or desire to switch  Receive x1 intragluteal injection for Apretude (600mg/ 3ml)  Liaise with peer navigator for follow-up |
| **M2** | Peer navigator adherence support | Peer support |
| **M3** | Peer navigator adherence support | HIV point of care testing x2 and dry blood spots for 4^th^ generation HIV antibody/antigen test, pregnancy test.  Contraception if needed and check happy / need to continue and or desire to switch  If safety bloods at M1 OK receive x1 intragluteal injection for Apretude (600mg/ 3ml)  Liaise with peer navigator for follow-up |
| **M4** | HIV point of care testing x2 and dry blood spots for 4^th^ generation HIV antibody/antigen test, pregnancy test.  Contraception if needed and check happy / need to continue and or desire to switch  (in choice arm)  Adherence support and liaise with peer navigator  Dispense 3-month supply of tenofovir disoproxil fumarate and emtricitabine (TDF/FTC) or DapiRing or PEP-in-pocket | Peer support |
| **M5** | Peer navigator adherence support | HIV point of care testing x2 and dry blood spots for 4^th^ generation HIV antibody/antigen test, pregnancy test,  Contraception if needed and check happy / need to continue and or desire to switch  Receive x1 intragluteal injection for Apretude (600mg/ 3ml)  Liaise with peer navigator for follow-up |
| **M6** | Peer navigator adherence support | Peer support |
| **M7** | HIV point of care testing x2 and dry blood spots for 4^th^ generation HIV antibody/antigen test, pregnancy test.  Contraception if needed and check happy / need to continue and or desire to switch  (in choice arm)  Adherence support and liaise with peer navigator  Dispense 3-month supply of tenofovir disoproxil fumarate and emtricitabine (TDF/FTC) or DapiRing or PEP-in-pocket | HIV point of care testing x2 and dry blood spots for 4^th^ generation HIV antibody/antigen test, pregnancy test,  Contraception if needed and check happy / need to continue and or desire to switch  Receive x1 intragluteal injection for Apretude (600mg/ 3ml)  Liaise with peer navigator for follow-up |
| **M8** | Peer navigator adherence support | Peer support |
| **M9** | Peer navigator adherence support | HIV point of care testing x2 and dry blood spots for 4^th^ generation HIV antibody/antigen test, pregnancy test, Contraception if needed and check happy / need to continue and or desire to switch  Receive x1 intragluteal injection for Apretude (600mg/ 3ml)  Liaise with peer navigator for follow-up |
| **M10** | HIV point of care testing x2 and dry blood spots for 4^th^ generation HIV antibody/antigen test, pregnancy test.  Contraception if needed and check happy / need to continue and or desire to switch  (in choice arm)  Adherence support and liaise with peer navigator  Dispense 3-month supply of tenofovir disoproxil fumarate and emtricitabine (TDF/FTC) or DapiRing or PEP-in-pocket | Peer support |
| **M11** | Peer navigator adherence support | HIV point of care testing x2 and dry blood spots for 4^th^ generation HIV antibody/antigen test, pregnancy test,  Contraception if needed and check happy / need to continue and or desire to switch  Receive x1 intragluteal injection for Apretude (600mg/ 3ml)  Liaise with peer navigator for follow-up |
| **M12** | Peer navigator adherence support | Peer support |
| **M13** | HIV point of care testing x2 and dry blood spots for 4^th^ generation HIV antibody/antigen test, pregnancy test, STI testing.  Contraception if needed and check happy / need to continue and or desire to switch (in choice arm)  Adherence support and liaise with peer navigator  Dispense 3-month supply of tenofovir disoproxil fumarate and emtricitabine (TDF/FTC)  Sexually transmitted infections, including POCT for syphilis, self-taken vaginal swabs or urine tests for gonorrhoea and chlamydia. | HIV point of care testing x2 and dry blood spots for 4^th^ generation HIV antibody/antigen test, pregnancy test,  Contraception if needed and check happy / need to continue and or desire to switch  Receive x1 intragluteal injection for Apretude (600mg/ 3ml)  Liaise with peer navigator for follow-up  Sexually transmitted infections, including POCT for syphilis, self-taken vaginal swabs or urine tests for gonorrhoea and chlamydia.  FBC, creatinine and LFT. |
| **M14** | Exit and refer to department of health or AHRI PrEP services | Exit and refer to department of health or AHRI PrEP services |


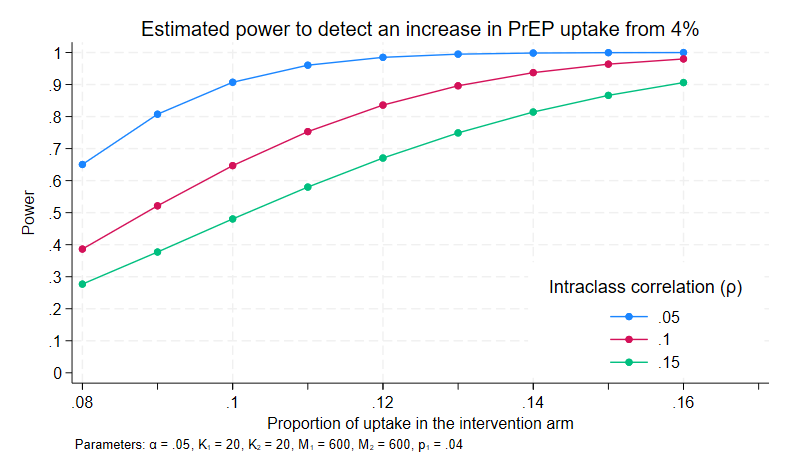


Supplementary Fig. 1 Power calculations assuming 4% PrEP uptake in the control arm


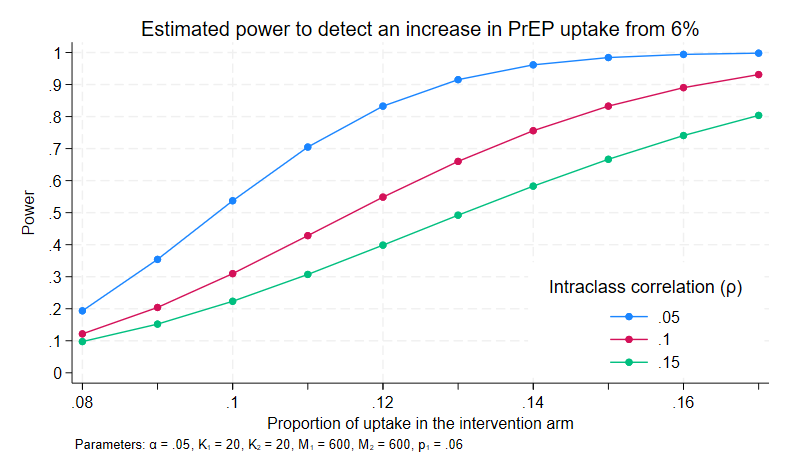


Supplementary Fig. 2 Power calculations assuming 6% PrEP uptake in the control arm
